# Transcriptional regulation on effector T cells in the pathogenesis of psoriasis

**DOI:** 10.1186/s40001-023-01144-0

**Published:** 2023-06-03

**Authors:** Yuying Qu, Dongmei Li, Huabao Xiong, Dongmei Shi

**Affiliations:** 1 College of Clinical Medicine, Jining Medical University, Jining, 272067 Shandong China; 2 Department of Microbiology and Immunology, Georgetown University Medical Center, Washington, DC USA; 3 Institute of Immunology and Molecular Medicine, Basic Medical School, Jining Medical University, Jining, 272067 Shandong China; 4 Department of Dermatology, Jining No. 1 People’s Hospital, Jining, 272067 Shandong China

**Keywords:** Psoriasis, T helper (Th) cells, Transcriptional regulation, Signaling pathway, Cytokine

## Abstract

Psoriasis is one of the most common inflammatory diseases, characterized by scaly erythematous plaques on the skin. The accumulated evidence on immunopathology of psoriasis suggests that inflammatory reaction is primarily mediated by T helper (Th) cells. The differentiation of Th cells plays important roles in psoriatic progression and it is regulated by transcription factors such as T-bet, GATA3, RORγt, and FOXP3, which can convert naïve CD4^+^ T cells, respectively, into Th1, Th2, Th17 and Treg subsets. Through the activation of the JAK/STAT and Notch signaling pathways, together with their downstream effector molecules including TNF-α, IFN-γ, IL-17, TGF-β, these subsets of Th cells are then deeply involved in the pathogenesis of psoriasis. As a result, keratinocytes are abnormally proliferated and abundant inflammatory immune cells are infiltrated in psoriatic lesions. We hypothesize that modulation of the expression of transcription factors for each Th subset could be a new therapeutic target for psoriasis. In this review, we will focus on the recent literature concerning the transcriptional regulation of Th cells in psoriasis.

## Background

Psoriasis, a chronic T cell-mediated inflammatory disease, affects almost 3% of the general population worldwide, and its incidence is increasing year by year [1]. The disease primarily involves skin, characterized by erythematosus plaque covered with silver-white scales [2]. The typical histological features of skin plaques include hyperproliferative keratinocytes in the epidermal layer and infiltration of T lymphocytes, dendritic cells, macrophages, and neutrophils in dermis layer.

The evidence has shown that different Th cells subtypes are involved in inflammatory responses of psoriasis [3–5]. The CD4^+^ T cells, helper T (Th) cells, can be differentiated to Th1, Th2, Th22/Th17 and Treg cells, play a central role in the pathogenesis of psoriasis. Each subset of Th1 cells above is particularly driven by an individual transcription factor such as T-bet, GATA-3, RORγt, or FOXP3. These transcription factors interact with the signal transducer and activator of transcription (STAT) protein to initiate various inflammatory signaling pathway, promoting the expression of downstream pro-inflammatory molecules such as interleukins, interferon-gamma (IFN-γ) and tumor necrosis factor-alpha (TNF-α). These pro-inflammatory cytokines are well known for their effects in pathogenesis of psoriasis.

**Fig. 1:**
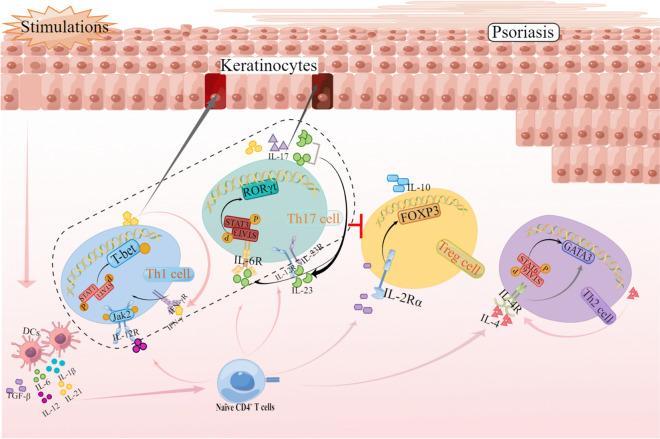
T cell transcriptional regulation mechanism in psoriasis. Psoriasis is driven by many nonspecific triggers. Triggers such as infections and physical injury stimulate DCs to release pro-inflammatory factors (IL-6, IL-1β, IL-21, IL-23, TNF-α and IL-12). These cytokines stimulate the activation of naive CD4 + T cells and secrete IL-12, IL-23 and IL-6, IL-10 and TGF-β, IL-4 differentiated into Th1 cells, Th17 cells, Treg cells and Th2 cells, respectively. IL-12 combines with naive CD4 + T cell surface receptor, IL-12R, and their interaction leads to activation of the transcription factors STAT1 and T-bet, thereby promoting the polarization of Th1 cells. T-bet and STAT1 form a positive feedback regulatory loop for upregulating IL-12 and promoting Th1 polarization. Th17 cells are differentiated by ROR-γt regulation is also regulated by JAK–STAT3 pathway. IL-6 produced in response to inflammation continuously activates the STAT3 pathway, which in turn induces IL-21 expression and forms an IL-21/STAT3 autocrine loop, thereby causing sustained activation of STAT3, upregulation of ROR-γt for Th17 differentiation. The differentiation of Th2 cells is regulated by GATA 3. The balance of Th1, Th17 and Th2 is an important factor affecting the development of psoriasis. Treg cells can suppress the activities of other immune effector cells, such as Th1 and Th17 cells, through either direct contact or the secretion of suppressive cytokines, such as IL-10 and transforming growth factor (TGF)-β. This figure was created with Figdraw.com

In this review, we focus on the literature that concerned the transcriptional regulation of Th cells and contribution of subsets on the pathogenesis of psoriasis. An imbalance between Th1 and Th2 cells in psoriasis was initially noted in an early study [6]. With new Th subsets identification, the impact of Th17 cells was increasingly recognized. In the chronic psoriatic lesions, infiltration is often dominated by Th17 cells. The differentiation of Th17 cells can not only be activated by IL-6 and IL-23, but also by Th1-associated cytokine IFN-γ. Furthermore, IFN-γ also stimulates the production of CCL20 from antigen-presenting cells (APCs), which is responsible for migration of the IL-17^+^ T cells [7]. Besides three effector Th subsets, the roles of regulatory T cells (Tregs) in psoriasis development have been noted and anti-inflammatory effect of Treg subset has been even considered as a treatment option for psoriasis [8, 9]. Transcription factor FOXP3 is required for Treg differentiation. Treg cells (CD4^+^CD25^+^) was first identified by Sakaguchi et al. in 1995, which has inhibitory effect on activation and proliferation of CD4^+^ T cell and CD8^+^ T cell [10]. Through suppression on effector lymphocytes such as Th17 cells [11], Tregs can inhibit pro-inflammatory responses and slow down tissue destruction. Thereafter, regulation network of transcriptional factors and signaling pathways for effector T cells (Th1, Th2 and Th17) and anti-inflammatory Treg response could be the key to elucidate underlying mechanism and develop the treatment strategies (Fig. 1).

### The interaction between transcription factors and STAT proteins regulates inflammatory response in the pathogenesis of psoriasis

The transcription factors (T-bet, GATA-3, RORγt, and FOXP3) for differentiation of each Th subset above interact with different STAT proteins to control downstream cytokine pathways.

#### The T-bet/Stat-1 axis regulates the differentiation of Th1 cells in the pathogenesis of psoriasis

Transcription factor T-bet regulates the transcriptional program of Th1 cells. T-bet, a member of the transcription factor T-box family, first reported in 2000. It contains 530 amino acids and a domain composed of 189 amino acids is responsible for binding to DNA T-box [12]. When T-bet primes differentiation of Th1 from naïve CD4^+^ T cell, it can inhibit the production of IL-4 (a Th2-associated cytokine) at the same time. As a result, there is a possibility to have a shift from Th2 to Th1 cell dominance in the inflammatory milieu.

T-bet can be induced in response to IFN-γ in a STAT1-dependent manner, that leads to remodel chromatin of the IFN-γ gene and stabilization of IFN-γ, to upregulate expression of IL-12Rβ and to activate IL-12-induced growth [13, 14]. IL-12 combines with naive T cell surface receptor, IL-12R, and their interaction leads to activation of the transcription factors STAT4 and T-bet, thereby promoting the polarization of Th1 cells. The level of IL-12 can be upregulated by secretion of IFN-γ from differentiated Th1 cells [15]. In addition, IFN-γ binding to IFN-γ receptor 1 and IFN-γ receptor 2, promotes phosphorylation of STAT1 at tyrosine and serine sites. The homo/heterodimers of p-STAT1 activate the expression of the gene T-bet that in turn promotes IFN-γ production by CD4^+^T cells and upregulates its own expression. Therefore, both T-bet and STAT1 form a positive feedback regulatory loop for upregulating IL-12 and promoting Th1 polarization [16–18]. Such conclusion was derived from the study, in which STAT1-deficient naive CD4^+^T cells show impaired Th1 differentiation and the Stat1^−/−^Th1 cells had reduced levels of IFN-γ and T-bet [19]. In psoriasis, this IFN-γ-STAT1-T-bet may also form a positive regulatory loop and plays a critical role in stabilizing Th1 phenotype. It has been shown that after treatment with TNF-α inhibitor in patients with psoriasis, serum levels of Th1-associated T-bet and related cytokines IFN-γ, IL-6 and TNF-α were significantly decreased when compared to the baseline period [20]. In animal model, mice with defective T-bet gene showed a decreased IL-12 expression in vivo and an attenuated type I immune inflammatory response [21]. A study has found that IL-17^+^ cells dominate the acute phase of inflammation, whereas T-Bet^+^ cells seem to increase gradually during the entire observation period [22], suggesting a likely more important role of IL-17 in the inflammatory stages and a more prominent role of T-Bet + cells at the chronic phase.

The Notch signaling pathway is critical for several cell activities, including cell proliferation and differentiation, particular in the differentiation of CD4^+^T cells [23–27]. Notch itself is a cell surface receptor and expression of Notch1 was shown to be upregulated in mouse psoriatic lesions along with its transcription factor RBPJK, Notch ligand JAG1 [28]. It has been reported that inhibition of Notch signaling prevents Th1 differentiation in vitro and in vivo, possibly by inhibiting the expression of T-bet [25]. Because T-bet can also be downregulated in RBPJK defective cells, the activation of Notch1 signaling could be also involved in promotion of Th1 responses.

Collectively, T-bet interacts with the STAT1 and perhaps the Notch pathways to regulate differentiation or warrant Th1 response in psoriasis.

#### The GATA-3/Stat-6 axis regulates Th2 differentiation and Th2-associated cytokines in psoriasis

GATA-3, a member of the GATA family of zinc finger proteins, contains 444 amino acids with a high homology among different species. GATA-3 protein is considered to be a T cell-specific transcription factor since it specifically binds to the enhancer of the four subunits of the T cell antigen receptor (TCR) to regulate TCR expressions [29]. Th2 cells develop from naive CD4^+^ T cells and are induced by IL-4 that is released by naïve Th cells, natural killer T cell (NK T cells), mast cells and activated Th2 cells. IL-4, together with IL-10 that are secreted by Th2 cells as well, act as anti-inflammatory effectors to suppress Th1 differentiation and production of Th1-associated IFN-γ and TNF-β, thereby attenuate the inflammatory response in psoriasis [30]. In addition to this Th1 inhibitory effect, the combination of IL-4 and IL-4R on naïve T cell surface not only further activates GATA3 for Th2 polarization, but also promotes expression of other transcription factors such as STAT6 and c-MAF.

STAT6 is the central mediator of the IL-4 signal pathways for Th2 development that has been approved by the fact that STAT6-deficient mice lack of Th2 differentiation [32]. In a study with 60 patients, RNA sequencing and protein networks analysis of lesioned area and normal skin areas found that GATA3 was significantly downregulated in psoriatic tissues [31]. Likely, the IL-4/STAT6 and IL-4/GATA3 signaling pathways prevent hyperactive Th1 responses in psoriasis. In a normal skin, GATA3 at a certain level can form a positive feedback to further upregulate its own expression and sustainably to inhibit Th1 differentiation [33, 34]. Phosphorylated IL-4R on the other hand also participate in recruiting and activation of STAT6. The activated STAT6 forming a dimmer that enters nucleus and in turn initiates the transcription of GATA3. Recently, a highly expressed miR-210 has been reported in CD4^+^ T cells at psoriatic lesions that was suggested to elicit immune imbalance and inflammatory responses in the peripheral blood and affected skin areas [35]. MicroRNA miR-210 has been known as a downstream target of HIF-1α and Th17- and Th1-associated inflammatory cytokines TGF-β and IL-23, which can inhibit Th2 differentiation through inhibition of STAT6 signal transduction pathway and function of tyrosine protein kinase.

The imbalanced Thl/Th2 has long been considered in explanation of psoriatic pathogenesis and treatment was then to reverse the imbalance. For example, the use of methotrexate to treat patients with psoriasis is based on that can significant reduce Th1-associated transcription factor T-bet, IL-12 and IFN-γ in serum, and increase Th2-associated GATA3 and IL-4 levels in patients with PASI 75 [36]. Therefore, when GATA3 gene was deleted, a complete suppression of Th2 cell differentiation led to exacerbation of psoriasis due to a predominantly Th1 response [38, 39]. Also, the expression of GATA-3 and the phosphorylation of STAT6 were specifically defective in cells having Th2 LCR-deficiency [37].

Notch acts in parallel with GATA-3 to synergistically activate IL-4 expression. GATA-3 is also a direct transcriptional target of the Notch pathway. Both GATA3 and STAT6 may act in concert with the Notch signaling pathway to control an optimal Th2 cell response during psoriasis [40], in which GATA3 is the central regulator for maintaining the effectiveness of Th2 response.

#### The STAT-3/RORγt axis regulates the differentiation of Th17 cells and Th17-associated cytokines in psoriasis

IL-17 producing Th17 cells are characterized by the expression of the transcription factor-retinoid-related orphan receptor gamma t(ROR-γt). They mediate inflammation through the secretion of IL-17A, IL-17F, IL-6, IL-21 and IL-22 [41]. RORγt plays a central role in Th17 differentiation [41, 42]. Since it has been identified in 2006, RORγt has received substantial attention in studying of Th17 responses in various inflammatory diseases [43]. It seems that naïve CD4^+^T cells are capable to carry on a self-differentiation process for Th17 and IL-17-mediated pro-inflammatory response via adequate expression of ROR-γt because T cells lacking ROR-γt failed to differentiate into Th17 [41]. ROR family is composed of three members, RORα (NR1F1), RORβ (NR1F2), and RORγ (NR1F3), encoded by Rora, Rorb, and Rorc genes, respectively [44–46]. Several transcription factors have been associated with function of RORγt regarding to Th17 response, including interferon regulatory factor 4 (IRF4), STAT3, Bob1, and basic leucine zipper transcription factor (BATF) [47, 48]. The resulting IL-17A is a particularly important effector molecule in the pathogenesis of psoriasis, since it inhibits keratinocyte differentiation and promotes their proliferation by downregulating Human Regenerating islet-derived protein 3-alpha (REG3A). At the same time, anti-microbial peptides produced by stimulating keratinocytes attract more inflammatory cell infiltration, thereby exacerbating the inflammatory response [49, 50]. IL-6 produced in response to inflammation continuously activates the STAT3 pathway, which in turn induces IL-21 expression and forms an IL-21/STAT3 autocrine loop, thereby causing sustained activation of STAT3, upregulation of ROR-γt for Th17 differentiation [51].

During the activated stage of STAT3, the transcription factors ROR-γt and RORα also induce the expression of IL-23R that sustains survival and stability of Th17 cells. Chan JR et al. found that direct intradermal administration of IL-23 in mouse skin can enhances the expression of IL-17A, however, pre-treatment with anti-IL-17A antibody cannot ameliorate psoriasiform lesion [52]. Obviously, IL-17A is not the only requirement for psoriasis pathogenesis, for example IL-17F can also bind IL-17RC [53]. IL-23 belongs to the IL-12 family that can bind to its receptors IL-12Rβ1 and IL-23R to enhance the activity of ROR-γt of Th17 cells. It also participates in the phosphorylation of GATA3 to show the impacts on the activation of keratin-forming cells and epidermal thickening associated with psoriasis [54]. IL-23 receptors are also be able to be induced by ROR-γt, leading to a Th17 polarization and production of IL-17A and IL-22, which further promote psoriasis [41, 55]. IL-6 secreted by dendritic cells is critical for the conversion of initial T cells to Th17 or Treg cells. In the absence of IL-6, primary T cells show more Treg properties [56]. For psoriasis, Treg abundance will deactivate Th17 response via their released cytokines such as IL-22, IL-17, and IL21, which ultimately inhibit epidermal keratinocyte proliferation [57, 58]. Of these inflammatory cytokines, IL-22 can also be secreted by Th-22 cells that are differentiated from primary T cells in the presence of TNF-α and IL-6 [59]. In psoriasis patients, IL-22 acts synergistically with IL-17, TNF-α and other cytokines to maintain the inflammatory response in psoriasis [60]. Moreover, IL-22 induces keratinocyte proliferation and epithelial proliferation, inhibits terminal epidermal keratinocyte differentiation, and thus induces epithelial psoriasis-like lesions [61, 62]. IL-22 has been known to upregulate the expression of various anti-microbial peptides from keratinocytes such as Human Beta-Defensin2 (HBD-2), Human Beta-Defensin3 (HBD-3) and S100A7. These anti-microbial molecules in fact could reduce the incidence of skin infections in patients with psoriasis [49].

Inhibition of the Notch1 signaling can disrupt Th17 cell differentiation and function through the downregulation of RORγt expression. In addition, the JAK/STAT signaling pathway transduces cytokine-mediated signals to prime CD4^+^ T cell differentiation. Although the JAK2/STAT3 pathway does not act on Th17 cells directly, it blocks upstream IL-23 for Th17 cell differentiation and then the cytokines [63].

Consistent with a key role of RORγt-dependent Th17 cells in autoimmunity, mRNAs for both RORγt and IL-17 were significantly increased in skin from psoriasis patients [64]. In this study, autoimmune inflammatory responses are somewhat reduced in psoriatic model of mice with RORγt gene defects [64].

#### The role of Foxp3 transcription factors in psoriasis

Treg cells are a special subset of helper T cells that are characterized by high expression of the CD25 (alpha-chain of IL-2 receptor). Foxp3 (Forkhead Box P3), a transcription factor of the fork head/winged-helix family, is the master transcription factor for Treg development and function. Treg cells can suppress the activities of other immune effector cells through either direct contact or the secretion of suppressive cytokines, such as IL-10 and transforming growth factor (TGF)-β [65]. In contrast to the function of Th17 cells, Treg cells inhibit the function of other lymphocyte subsets, which ultimately suppresses immune responses, inflammation, and tissue destruction [66].

A latest study has discovered that dysfunctional Tregs from peripheral blood of psoriatic patients show phosphorylation of the STAT3 pathway as well as high expressions of the pro-inflammatory cytokines IFN-γ, TNF-α and IL-17A [67]. Apparently, immune regulation for an increased Th17 cells and a decreased Treg cells would increase pathogenic impacts on psoriasis development. For example, IL-17A can block the suppressive function of Tregs, possibly by inhibiting the release of TGF-β and promoting the production of IFN-γ. The latest study has confirmed this view [68]. On the other hand, upregulation FOXP3 and downregulation of RORγt expression inhibited Th17 cell differentiation and restored Treg function. Based on these observations, treatment strategies for psoriasis could be restoration of Treg function or repair of the Th17/Treg cell imbalance [8]. Indeed, after treatment with IL-17A inhibitor, a significant reversal of the Th1/Th17 activation and a concomitant upregulation of Th2 and Treg subsets were found [69], which were demonstrated by decreased expressions of T-bet and RORγt and by increased expressions of GATA3 and FOXP3.

### Macrophage-related transcription factors in psoriasis

Innate immunity in the skin provides the first line of defence against infection and plays a pivotal role in regulating subsequent adaptive immunity in infectious disease and inflammatory diseases. Numerous studies have shown that there is difference of infiltrating immune cells between progressive and stable phases of psoriatic lesions. At progressive stage, there are a large number of macrophages in affected skin lesions [70]. Since a selective elimination of macrophages can improve psoriasis lesions in mice, macrophages seem have a key role in the pathogenesis and progression of the disease [71]. Today, macrophages have been classified into different subtypes (M1, M2a, M2b and M2c) according to activation of their surface markers and functional capabilities. It has been shown that peripheral monocytes in psoriasis patients tend to undergo more M1 polarization than M2 polarization; patients with severe psoriasis had a higher ratio of M1/M2 macrophages [72]. Once activated, TNF-α secreted by macrophages activates STAT1 of the JAK/STAT signaling pathway, which further polarizes macrophages toward M1, promotes the development of inflammation, secretes TNF-α, IL-6, IL-20. The exaggerated M1 cells favor the development of Th1 and Th17 immunity, causing keratin-forming cells to proliferate and aggravating psoriasis [73]. The role of interferon regulatory factor (IRF)-1 in M1 polarization has also been demonstrated in recent studies [74, 75]. The transcriptional regulator IRF-1, which is weakly expressed on macrophages at rest, can be robustly upregulated by IFN-γ, also aggravates psoriasis.

### Transcription factors as new therapeutic targets of psoriasis

Transcription factors form the basis of selective immune response control and are one of the most promising but underutilized classes of drug targets [76]. As we described above, 4 transcription factors for each major lineage of Th cells interact each other and with other signaling molecules to influence the inflammatory process in psoriasis. Unsurprisingly, modulating transcriptional disorder is a common objective in many drug discoveries. In regard to transcriptional factors, the act of attenuating psoriatic inflammatory cascade would be accomplished by modulating the transcription factors for differentiation of the Th1, Th17, Th22 and Treg cells [77].

The early studies in mouse psoriasis models and clinical trials have simultaneously demonstrated that immune reagents targeting cytokines, for example, IL-17 antagonists or other cytokines upstream and downstream of the inflammatory response, could effectively reverse psoriasis [78]. Given the importance of RORγt in the differentiation of Th17 cells, a potent and selective RORγt antagonist (A213) was approved to attenuate psoriatic lesions in two different mouse models by inhibiting the production of IL-17 and Th17 cell differentiation [79, 80]. With the same token, a small Stat3 inhibitor, STA-21, was found to ameliorate psoriatic flares not only in mice, but also in humans [81], which was based on the fact that higher levels of inflammatory cytokines and growth factors promote Stat3 activation in epidermal keratinocytes in psoriatic lesions. Suppression of Th1 responses also resulted in a reduced expression of T-bet [82] that may be through inhibiting IFN-γ-induced STAT1 and IL-12-induced STAT3/STAT4 activation. PSORI-CM02 was found to upregulate the expression of GATA3 in psoriatic lesions, resulting in an improvement on psoriatic lesions in mice, but had no effects on T-bet or RORγt responses [83]. Finally, any strategy to increase FOXP3 expression would increase Treg cells and function, thereby improving psoriatic lesions [84].

### Single-cell transcriptomic analysis of psoriatic T cell populations

Previous studies on transcriptional differences between psoriasis lesions and control groups have been based on traditional RNA sequencing, which includes the overall transcriptional differences of all cells in tissue samples. Therefore, response of specific immune cells are often masked and diluted by a large amount of keratinocytes [85, 86]. In recent years, the development of single-cell sequencing technology (scRNA-seq) has overcome these limitations. Through subclustering the cell populations, single-cell expression profiles enable a detailed analysis of heterogeneity and interrelationships [87]. ScRNA sequencing has been applied in diagnosis and treatment of inflammatory skin diseases.

For psoriasis, the scRNA-seq analysis has identified an aberrant inflammatory transcription of A20 in KCs of psoriasis, that is related to the IL-17 and TNF-α signaling pathways and may have potential for targeted therapy [88]. This technique also helped to identify a CD4^+^ Treg cell population in psoriasis that was noted by the expression of FOXP3, TIGIT, CTLA4, IL2RA (CD25), and IKZF2. There are two cytotoxic T cell clusters (CD8A^+^CD8B^+^), both express CCL5, GZMB, and NKG7 and was classified as exhausted T cells (CTLex) [89]. In psoriatic skin, scholars have discovered 11 CD8^+^T cell clusters through single-cell sequencing technology. Among them, clusters 0, 4, 6, 7, and 10 were enriched in psoriatic lesions. These included the Tc1-like cluster 9 that highly expressed the Type I cytokine genes IFN-γ and TNF. Cluster 6 and 10 were identified as two Tc17 subsets based on high expression of IL-17A and IL-17F, these two clusters could also express CXCL13 [90]. Even the cutaneous type 17T (T17) cells showed different transcriptome profiles on cytokines IL-17A, IL-17F and IL-10 [91–93]. Upregulation of CXCL13 in psoriatic lesions was also observed in a previous bulk RNA-seq study [94] and it was positively correlated with psoriasis severity.

## Conclusion and perspective

Psoriasis is an inflammatory disease associated with Th cells, and pro-inflammatory factors regulate the differentiation of T cells and recruit inflammatory cells through relevant signaling pathways, leading to abnormal inflammation and abnormal cell proliferation. While targeted therapy with psoriasis biologics targets specific cellular components and cytokines in the inflammatory pathway has made a lot of progress, there are still problems of drug failure and disease recurrence, and may cause dysregulation of other pathways. Currently, there seems to be no biological agents that directly target transcription factors; however, some research suggests that T cell transcription factor levels can serve as markers for detecting the severity and activity of psoriasis. Some scholars have found that transcription factors that promote psoriasis are highly expressed throughout the entire course of the disease, so in the chronic phase, targeted transcription factors are expected to become a new treatment. In the future, we will use scRNA-seq technology to more accurately identify drug targets and make personalized medication a reality.

## Data Availability

Not applicable.
